# GenBlosum: On Determining Whether Cancer Mutations Are Functional or Random

**DOI:** 10.3390/genes17010055

**Published:** 2026-01-02

**Authors:** Alejandro Leyva, Muhammad Khalid Khan Niazi

**Affiliations:** 1 Department of Biomedical Engineering, The Ohio State University, 2255 Kenny Rd., Colombus, OH 43210, USA; leyva.29@osu.edu; 2 Department of Pathology, The Ohio State University, 2255 Kenny Rd., Colombus, OH 43210, USA

**Keywords:** somatic mutation, codon-aware model, BLOSUM, neutral evolution, cancer genomics, TP53, PIK3CA, Monte Carlo simulation

## Abstract

Background: Genetic mutations have proven to be the epicenters of cancer and disease progression. Traditional WXS sequencing and BLOSUM scoring can be used to infer the evolutionary conservation of amino acid substitutions, though these approaches are not informed by probable base pair sequence changes. Within gene mutation analysis, most tools focus on amino acid conservation or codon switching independently, limiting their ability to contextualize observed mutations against stochastic mutational processes. In the clinical setting, variants of unspecified significance remain difficult to interpret, as clinicians are often unable to determine whether observed mutations arise from oncogenic selection or from stochastic mutational degradation. Methods: We analyzed mutation sequences from the TCGA BRCA cohort for TP53 and PIK3CA and developed a model that integrates BLOSUM scoring with statistical modeling of base pair changes to evaluate deviation from codon-aware neutral expectations. Observed mutational distributions were compared against a stochastic neutral model to assess statistical significance. Results: Within the TCGA BRCA cohort, TP53 mutations were significantly more evolutionarily radical than expected under the codon-aware neutral model, while PIK3CA mutations were significantly more evolutionarily conservative, as determined using chi-square testing. These opposing patterns are consistent with the distinct functional roles of TP53 and PIK3CA in oncogenesis, where TP53 is inhibited through disruptive loss-of-function mutations, whereas PIK3CA is recurrently mutated in a manner that preserves protein structure and promotes constitutive pathway activation. This contrast reflects selective pressure toward disabling tumor suppressor function while maintaining persistent oncogenic signaling. Conclusions: Codon-aware neutral modeling provides a statistical framework for distinguishing mutations that deviate from stochastic expectations and may aid in the interpretation of variants of unspecified significance. By contextualizing mutational severity relative to neutral processes, this approach offers insight into tumor evolution and may support prognostic assessment without relying on predefined gene-level neutrality.

## 1. Introduction

Genetic mutations are classified into missense, nonsense, and splice-site mutations, each of which has significant mechanistic effects on disease progression. Missense mutations are genetic mutations whereby the codon sequence for an amino acid is changed to code for another amino acid, whereby the change in amino acid induces functional differences that can result in dysregulation or dysfunction. Nonsense mutations are mutations that result from changing a codon to code for a START or STOP sequence that results in half-formed or dysfunctional proteins. Splice-site mutations occur when the intron regions of the coding sequences are appended or rearranged within the sequence, which can add amino acids that change the composition and folding of the protein. Within breast cancer, key mutations include KRAS, which controls the regulation of downstream processes and uptake from cellular receptors [1]. TP53 (tumor suppressing antigen 53) is a protein that regulates apoptosis, activation of EMT and PI3KT, and serves as a transcription factor [2,3,4]. Within tumors, TP53 is mutated to suppress apoptotic signals or regulation of proliferative pathways by binding to the DNA-binding domain of the protein to decouple the protein’s ability to bind to DNA [2,3,5]. PIK3CA is a tumor gene that activates PIP2, which activates the AKT pathway, thereby suppressing apoptotic signals, and is used to constitute a cycle of proliferative signals [6,7,8,9].

These mutations can be random and a result of double strand breaking or other chronic diseases, and the likelihood of a mutation being random can be evaluated using evolutionary conservation models [10,11,12,13]. Within missense mutations, if an amino acid from a chemical group is substituted with an amino acid from another chemical group, the likelihood that the tumor is random depends on the likelihood that the codon change is able to occur. Certain codon switches, such as point mutations in osteogenesis, require a change in a single codon base to translate. Other base changes require the substitution of several codons, which is statistically less likely to be observed using stochastic models [14,15]. BLOSUM scoring provides an established measure of evolutionary conservatism for amino acid substitutions, which in this study is interpreted in the context of codon-aware mutational probabilities rather than direct nucleotide-level changes [16,17]. Because BLOSUM does not explicitly model the probability of codon-level nucleotide substitutions, this motivates integrative approaches that jointly consider mutational likelihoods and biochemical consequences of amino acid changes.

Tools such as PolyPhen and SIFT evaluate mutations using evolutionary conservation and amino acid chemistry, but do not explicitly model codon-level nucleotide substitution probabilities [18,19,20,21]. Measures such as dN/dS quantify synonymous and non-synonymous substitution rates, but do not capture the biochemical severity or structural consequences of amino acid changes [15]. Models based on mutational signatures, such as APOBEC, characterize the processes shaping genomic mutation patterns but do not directly address the functional impact of resulting protein alterations [22]. As cancer genomes are shaped by heterogeneous and non-uniform mutational processes, comprehensive interpretation of somatic mutations requires integration across genetic, evolutionary, and biochemical levels.

Within the clinic, tumor gene panels such as BRCA variants of unknown significance (VUS) are used to assess mutations in DDR genes, which infer tumor aggressiveness and lower prognosis, as well as resistance to traditional chemotherapy regimens like FOLFIRINOX [23,24]. As oncology moves toward mutation-based therapies, there is an increasing need to evaluate the functional significance of the mutation across genetic and biochemical levels. In this study, we establish a neutral model that accounts for the probability of genetic mutations within a cancer cohort, as well as accounting for the evolutionary phylogeny and biochemistry of amino acid changes. Using large samples derived from Whole Exome Sequencing (WXS), we can use cohort-level analyses of TP53 and PIK3CA mutations to determine the statistical significance of amino acid substitutions based upon simulations of codon changes, to produce a neutral distribution of BLOSUM scoring to compare against the observed distribution.

While traditional codon substitution models, such as that of Goldman and Yang [25], focus on the stochastic evolution of germline sequences under long-term evolutionary assumptions, the present work addresses a different problem: assessing neutrality in somatic cancer mutations. Cancer evolution violates many assumptions underlying classical codon models due to context-dependent mutational processes, clonal selection, and non-equilibrium dynamics. Consequently, we employ a novel, empirical, and cohort-specific neutral framework to evaluate whether the severity of observed amino acid substitutions deviates from expectations under stochastic somatic mutagenesis. Rather than replacing existing codon models, our approach complements them by extending neutrality testing into the somatic cancer setting.

## 2. Materials and Methods

1000 WXS samples were processed from TCGA BRCA [1], and all mutations were compared against the base DNA sequence for TP53 from the UNIPROT p04637 FASTA [2,3,4]. If a missense or nonsense mutation was detected for TP53 or PIK3CA, all amino acid sequences were recorded by the program, and each mutational sequence was placed into a separate FASTA file along with a spreadsheet containing the amino acid sequence. The coding sequences for TP53 and PIK3CA were used to construct the neutral observation model [11,12]. Each codon was annotated in the sequence using HGVSp labels (e.g., Arginine 273 is R273), and the sequences of mutations were recorded per case, whereby a minority of cases had more than one biospecimen that resulted in another mutation set. Each missense and nonsense protein is applied to the original WT sequence and then abridged into the CSVs for each case. The UNIPROT WT coding sequences were applied to the statistical model, in addition to the WT amino acid sequences [2,3].

To construct the neutral model, each position and each mutation type were analyzed and taken from the observed distribution of mutations [11,12,13]. The likelihood of the nucleotide changes in the codon sequences was evaluated using probabilistic weights derived from prior literature describing somatic mutational processes consistent with breast adenocarcinoma [14,15]. Classical codon models employ Ti/Tv ratios as low-dimensional summaries to enable parameter estimation under equilibrium assumptions, whereas cancer mutation modeling often benefits from empirically derived substitution probabilities that reflect context-dependent somatic processes [25]. Monte Carlo simulations were performed to produce a stochastic analysis of all mutations that occur at that position as encoded. The probabilities were summed, and the amino acid changes resulting from the codon changes were used to construct a BLOSUM distribution [16]. The observed distribution of BLOSUM scores across all positions was tested via a Monte Carlo one sided tests against the neutral model’s distribution of mutation observations after 7600 Monte Carlo simulations for convergence testing.

## 3. Statistical Model

We begin with encoding the codon space into groups of three, whereby all coding sequences are divisible by three, allowing appropriate translation and positional tracking in amino acid sequences. The likelihood of an amino acid change is the likelihood of the nucleotide change at the position, which is contextually dependent and based on prior literature for breast adenocarcinoma [1,11,12,13]. Monte Carlo simulations are taken to observe the stochastic distribution of base changes, and thus amino acid changes, at all codons that were mutated in the observed distribution [14,15]. Because the neutral distributions are generated via Monte Carlo simulation, consistency was verified via replicate simulations.

## 4. Model Parameters


$$\begin{array}{cc} L & :\;number\;of\;codons\;in\;the\;gene\;CDS\\ c_{i} & :\;wild–type\;codon\;at\;position\;i,\;c_{i}\in \{A,C,G,T{\}}^{3}\\ a_{i} & :\;wild–type\;amino\;acid\;at\;position\;i,\;a_{i}=T(c_{i})\\ E & :\;set\;of\;all\;single–nucleotide\;missense\;events\;(no\;STOP)\\ J & :\;total\;number\;of\;neutral\;missense\;events,\;|E|=J\\ A_{j},A_{j}^{\prime } & :\;WT\;and\;neutral\text{-}mutant\;amino\;acids\;for\;event\;j\\ S_{j} & :\;BLOSUM62\;score\;for\;event\;j,\;S_{j}=B(A_{j},A_{j}^{\prime })\\ R_{j} & :\;radical\;indicator\;for\;event\;j,\;R_{j}=1\{S_{j}\leq 0\}\\ w_{b\to b^{\prime }} & :\;base\text{-}substitution\;weight\;for\;b\to b^{\prime }\\ {\tilde{p}}_{j} & :\;raw\;neutral\;weight\;for\;event\;j\\ p_{j} & :\;normalized\;neutral\;probability\;of\;event\;j,\;p_{j}=\frac{{\tilde{p}}_{j}}{\sum_{m=1}^{J}{\tilde{p}}_{m}}\\ N & :\;number\;of\;observed\;missense\;mutations\;from\;the\;cohort\\ S_{i}^{\mathrm{obs}} & :\;observed\;BLOSUM\;score\;for\;cohort\;event\;i\\ R_{i}^{\mathrm{obs}} & :\;radical\;indicator\;for\;cohort\;event\;i,\;R_{i}^{\mathrm{obs}}=1\{S_{i}^{\mathrm{obs}}\leq 0\}\\ \mu_{\mathrm{obs}} & :\;observed\;mean\;BLOSUM\;score,\;\mu_{\mathrm{obs}}=\frac{1}{N}\sum_{i=1}^{N}S_{i}^{\mathrm{obs}}\\ r_{\mathrm{obs}} & :\;observed\;radical\;fraction,\;r_{\mathrm{obs}}=\frac{1}{N}\sum_{i=1}^{N}R_{i}^{\mathrm{obs}}\\ M & :\;number\;of\;Monte–Carlo\;neutral\;replicates\\ \mu_{s} & :\;neutral\;mean\;BLOSUM\;for\;replicate\;s\\ r_{s} & :\;neutral\;radical\;fraction\;for\;replicate\;s\\ p_{\mathrm{mean}} & :\;one–sided\;p–value\;for\;mean\;score,\;p_{\mathrm{mean}}\approx \frac{1}{M}\sum_{s=1}^{M}1\{\mu_{s}\leq \mu_{\mathrm{obs}}\}\\ p_{\mathrm{rad}} & :\;one–sided\;p–value\;for\;radical\;fraction,\;p_{\mathrm{rad}}\approx \frac{1}{M}\sum_{s=1}^{M}1\{r_{s}\geq r_{\mathrm{obs}}\} \end{array}$$

**Neutral model:** CDS describes the codon sequences, where the coding sequences are split into three base pairs each for codons, and within codons are set c, containing each base.

$${CDS}_{g}=(c_{1},\ldots ,c_{L}),\;c_{i}\in \{A,C,G,T{\}}^{3},$$

The likelihood of amino acid shift a is dependent on the likelihood of the base pair change in that sequence based on contextual analysis. The likelihood of one base shift is not the same as another base pair shift.

$$a_{i}=T(c_{i}),\;i=1,\ldots ,L,$$

Across a codon sequence, there are a total of 9 permutations possible, which are probabilistically weighted based on the probability of established base pair changes. Within those permutations, nonsense mutations are excluded, and the sequences of the WT and mutated samples must have different codons.

$$E=\{(A_{j},A_{j}^{\prime }):A_{j}=a_{i},\;A_{j}^{\prime }=T(c_{i}^{\left(k,b^{\prime }\right)}),\;A_{j}^{\prime }\neq A_{j},\;A_{j}^{\prime }\neq \mathrm{STOP}{\}}_{j=1}^{J}.$$

This results in the formation of set E of codon missense mutations possible, which are summed probabilistically to produce the likelihood of an amino acid shift in the codon. This likelihood is taken against the likelihood of any other mutation at that codon. **Signature weights:**

$${\tilde{p}}_{j}=w_{b\to b^{\prime }},\;p_{j}=\frac{{\tilde{p}}_{j}}{\sum_{m=1}^{J}{\tilde{p}}_{m}},\;j=1,\ldots ,J.$$

BLOSUM scores reflect evolutionary constraints on amino acid substitutions rather than direct functional or biochemical impact; however, systematic deviation of observed BLOSUM score distributions from codon-aware neutral expectations, particularly when aggregated across functional domains, provides evidence of selective pressures associated with oncogenesis.

**BLOSUM scores under neutral:**

$$S_{j}=B(A_{j},A_{j}^{\prime }),\;R_{j}=1\{S_{j}\leq 0\}.$$

Within the observed set, each missense mutation is established as a variant of the WT, and the likelihood of each missense mutation in the cohort is evaluated by the total observations of a specific mutation in a sample. BLOSUM scores B are computed for each mutation in the set, and all BLOSUM scores that fall below 0 are counted as radicals. The fraction of radical samples, or likely evolutionary divergent mutations, can be determined.

**Observed missense set:**

$$\{(a_{i}^{\mathrm{wt}},a_{i}^{\mathrm{mut}}){\}}_{i=1}^{N},\;S_{i}^{\mathrm{obs}}=B(a_{i}^{\mathrm{wt}},a_{i}^{\mathrm{mut}}),\;R_{i}^{\mathrm{obs}}=1\{S_{i}^{\mathrm{obs}}\leq 0\}.$$

$$\mu_{\mathrm{obs}}=\frac{1}{N}\sum_{i=1}^{N}S_{i}^{\mathrm{obs}},\;\;r_{\mathrm{obs}}=\frac{1}{N}\sum_{i=1}^{N}R_{i}^{\mathrm{obs}}.$$

Across the neutral distribution, Monte Carlo simulations are performed to produce random mutations and record their likelihood, BLOSUM score them, and then record the fraction of radical mutations in the distribution. The mean BLOSUM score of a mutation is evaluated by the summation of BLOSUM scores against the total population. **Monte Carlo neutral replicates:**

$$j_{k}^{\left(s\right)}∼Categorical(p_{1},\ldots ,p_{J}),\;k=1,\ldots ,N,\;s=1,\ldots ,M,$$

$$\mu_{s}=\frac{1}{N}\sum_{k=1}^{N}S_{j_{k}^{\left(s\right)}},\;r_{s}=\frac{1}{N}\sum_{k=1}^{N}1\{S_{j_{k}^{\left(s\right)}}\leq 0\}.$$

*p*-values are computed using the fraction of radical amino acid changes across both distributions, and the observed BLOSUM distribution across each sample. All reported *p*-values were derived from empirical Monte Carlo procedures based on codon-aware neutral simulations. We test whether observed mutations are more evolutionarily radical than expected under neutrality.

**One-sided** p**-values:**

$$p_{\mathrm{mean}}\approx \frac{1}{M}\sum_{s=1}^{M}1\{\mu_{s}\leq \mu_{\mathrm{obs}}\},\;p_{\mathrm{rad}}\approx \frac{1}{M}\sum_{s=1}^{M}1\{r_{s}\geq r_{\mathrm{obs}}\}.$$

Using this model, the likelihood that a mutation is functional can be evaluated by taking into account the likelihood of a codon change and the evolutionary conservatism of that change.

## 5. Results

Across 204 TP53 mutations and 172 mutations for PIK3CA, the percentage of radical mutations comprised the majority of mutations in the neutral distribution based on the positions of the mutations within the observed distributions in Table 1. The radical fraction of PIK3CA mutations was drastically lower than the radical fraction of the neutral model, while the TP53 radical fraction was indeed much higher than the neutral model [4,5,7,8,9]. The mean BLOSUM scores for the neutral distributions suggest that most mutations were relatively less evolutionarily conserved, though not at an extremity [16]. The observed mean BLOSUM score for the PIK3CA mutations was lower than the neutral distribution, inferring higher conservatism, while TP53 had a lower observed mean BLOSUM score [26,27,28]. The agglomerated radical fractions and BLOSUM scores across all distributions resulted in a higher radical fraction than all distributions separately shown in Table 1. Consequently, the average BLOSUM score was also lowest in the agglomerated distributions.

Chi-square testing was performed across mutations within all domains of each protein to determine the statistical significance and deviation from the neutral model shown in Table 2. Across domains, each deviation from the neutral model across both genes was statistically significant. The codon-aware stochastic neutral model serves as the negative control in this analysis, as gene-level neutrality cannot be assumed in cancer contexts. The deviation from the neutral model was extremely high, while the significance across domains was low. The null hypothesis is rejected that these mutations occur randomly within the TCGA-BRCA cohort [1,11,12]. The G statistic suggests that the distribution of mutations across both genes is extremely variant across the stochastic null model, which is supported by radical fractions within both genes [4,5,7,8,9,16]. TP53 observed a lower evolutionary conservatism score than the neutral model, while PIK3CA had a higher evolutionary conservatism score [26,27,28]. Across domains, the mutation distribution varies highly from the neutral mutational model.

Across protein domains, the distribution of protein domain mutations shown in Table 3 presents a lower variability in mutation domain location in the observed distribution over the neutral model. More mutations were observed in the Helical and Kinase domains within the observed sample cohort [7,8,9,27,28,29,30]. The neutral model presented a wider range and representation of domain mutations, modeling stochastic enrichment patterns. The enrichment compared in both distributions results in a 229% increase in enrichment of the Helical domain and a 130% increase in the Kinase domains. The enrichment of the remaining domains was fractions of the neutral model, similar mutational enrichment is reported in literature [31,32]. The BLOSUM scores within each domain were much lower within the neutral model than within the observed, with all of the BLOSUM mean scores for PIK3CA being lower than the neutral model, supported by lower radical fraction and lower average BLOSUM score across the entire protein [16].

Table 4 presents the domain enrichment for the TP53 gene, where almost all mutations observed in the distribution occur in the DNA-binding domain (DBD), as opposed to the dimerization, transcriptional activation, and ubiquitination domains [2,3,4,5]. In contrast, the neutral model has an equitable distribution of mutations across all domains within the protein. Lower enrichment of all domains within the TP53 protein was observed, with the exception of 220% enrichment in the DBD. The neutral BLOSUM mean scores were fairly low, which is anticipated since most evolutionarily conservative mutations are less likely to occur within missense mutations [16]. The observed BLOSUM mean within the TP53 protein was dramatically lower in the DNA-binding domain and oligomerization domain, while the remaining domains had no mutations [2,3,4].

**Figure 1:**
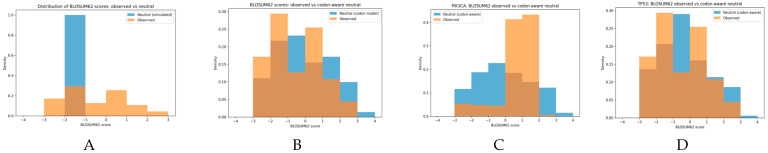
BLOSUM sBcore distributions for observed and neutral missense mutations. From left to right: (**A**) Observed versus neutral BLOSUM score distribution. (**B**) Codon-aware neutral BLOSUM distribution. (**C**) BLOSUM scores for *PIK3CA* missense mutations. (**D**) BLOSUM scores for *TP53* missense mutations.

**Figure 2:**
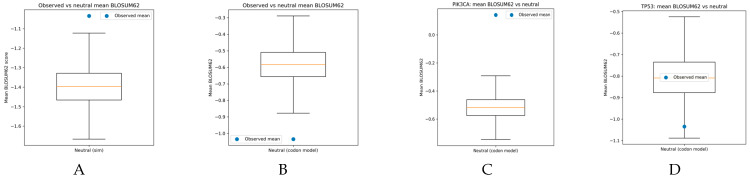
Mean BLOSUM scores for observed vs. neutral missense mutations. From left to right: (**A**) Mean BLOSUM score per gene shown as a boxplot. (**B**) Mean BLOSUM score for the codon-neutral background. (**C**) Mean BLOSUM score for PIK3CA. (**D**) Mean BLOSUM score for TP53. The orange line indicates the median BLOSUM score of the neutral distribution.

Within Figure 1A, a non–codon-aware neutral model was used to compare the distribution of observed and neutral BLOSUM scores, which resulted in the majority of scores secluded between −2 and −1, while the observed BLOSUM distribution across both proteins was relatively well dispersed [16]. In Figure 2B, the codon-aware model had a unimodal distribution of BLOSUM scores compared to the observed model in both genes, which is due to the evolutionarily conserved PIK3CA [7,8,9,27,28,29,30]. In Figure 2C, the PIK3CA distribution of BLOSUM scores was centered in the 0–2 range, suggestive of conservative base changes, while the neutral model exhibits a wider and lower distribution. The TP53 distribution in Figure 2D is bimodal in comparison to the neutral model, though there is a higher frequency of radical scores within the observed model [4,5,26,27]. This suggests alignment with BLOSUM scoring observed in protein domains and apt stochastic modeling of gene mutations [16].

The box plots for the distribution of scores for the neutral model and the observed mean are compared across both genes and across the control and codon-aware models. While this study focuses on TCGA BRCA and well-studied oncogenes, the framework is cohort-agnostic in principle and could be applied to other cancer types using cancer-specific neutral models. Figure 2A demonstrates complete misalignment with the observed mean of the two genes, with limited variability. Figure 2B presents a wider range of values that are less radical, though the observed mean is much lower across both genes [16]. This presents the alignment with distributions observed in Figure 1B. PIK3CA observed means in Figure 2C were observed to be more evolutionarily conservative, and the exact percentiles of the distributions are observed in the neutral model, and there is significant variability between the neutral and observed models [7,8,9,27,28,29,30]. In Figure 2D, the observed mean is significantly lower than the observed distribution, suggesting inhibition of the DNA-binding domains that regulate EMT and PI3K signaling [2,3,4,5,26].

**Figure 3:**
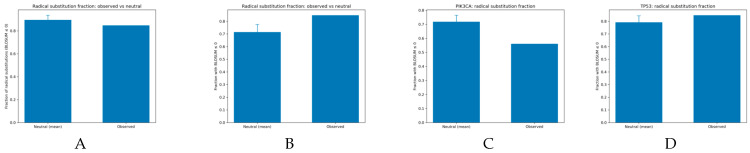
Radical and conservative missense mutation fractions in observed and neutral contexts. From left to right (**A**) Radical versus conservative mutation fraction across all genes. (**B**) Codon-neutral radical-to-conservative mutation fraction. (**C**) Radical mutation fraction for *PIK3CA*. (**D**) Radical mutation fraction for *TP53*. Whole-exome sequencing across the cohort revealed a high burden of TP53 mutations, whereas PIK3CA exhibited a more conservative mutation profile than typically observed in traditional tissues [7,8,9]. This presents an understanding of functional oncogenesis that can be used for further evaluation across cohorts. The high conservation of PIK3CA presents a functional understanding of tumor evolution and expansion, given the role of PIK3CA in the transduction to mTOR and AKT using PIP2 [6,26,27,28,30]. TP53 mutations focus on defunctionalizing by crippling the DNA-binding domain or by weakening the oligomerization domain such that other antigens cannot bind [2,3,4,5]. The coordination with zinc ions within the DNA-binding domain is disrupted, preventing the transcription of genes that upregulate apoptosis signals, including NOXA [2,3,4].

The observable radical fractions in Figure 3A are similar in the control non–codon-aware model, where there is a higher radical fraction in the neutral model than in the observed model, likely skewed by PIK3CA [7,8,9,27,28,29,30]. Within the codon-aware model, there is a lower radical fraction as compared to the observed distribution, which accounts for stochastic base-pair changes within the codon [14,15,16]. In Figure 3C, the radical fraction is observed to be lower using the codon-aware model, with lower variability than the neutral model. In Figure 3D, the radical fraction is higher in the observed for TP53, which supports the distributions shown in Figure 2 and the values in Table 1 [4,5,26]. Overall, the codon-aware model presents a higher variability of amino acid changes that can measure the deviation of genes from stochastic processes [11,12,16].

PIK3CA’s concentration of mutations within the Helical and Kinase domains represents the functional purpose of removing inhibition and catalyzing the function of PIK3CA for activation of proliferative pathways [7,8,9,27,28,29,30]. Typical mutations within the Helical domains focus on weakening the affinity of the catalytic core to the p85 antigen that regulates the affinity to RAS-GTP, which in turn activates the PI3K pathway [26,27,28]. These mutations are typically substitutions with glutamic acid that increase the affinity for RAS-GTP and occur in residues 200–275 [27,28]. Mutations within the Kinase domain focus on catalyzing protein activity, typically at H1047, creating an intrinsically active protein by stabilizing the active conformational state [26,27,28].

Functionally, the deviation from the neutral model provides insights into activity and evolution within malignant epithelial cells [11,12,13]. The neutral model provides potential for the prediction of statistically significant variations in mutational burden as compared to stochastic processes. Clinically, this provides potential avenues of prognosis prediction, chemosensitivity prediction, disease progression analysis, and internal assessments of symptom progression using different gene mutations. Genetic variation and mutation within samples can be studied to understand the progression of tumor evolution and statistically predict the likelihood of hallmarks of disease progression, squamous differentiation, and necrosis. Clinically, traditional software and annotation tools can provide more grounded assessments for diagnosis [18,19,20,21,23,24].

## 6. Limitations

This study is limited by the sample size of the cohort as well as the number of mutations within each cohort. For future work, multiple cohorts will need to be tested across several oncogenes to determine whether the model can sufficiently generalize across cancers. Because the model evaluation is dependent on the availability of mutations, there is no guarantee of demographic diversity, nor that there is a diversity of mutations across each stage of a tumor. The study did not account for general demographics, cancer stage, and other covariates. The study does not generalize across other cancers and only concludes relevance for breast adenocarcinoma.

In addition, BLOSUM scoring reflects the evolutionary conservation of amino acid substitutions and is not necessarily indicative of direct functional impact. While evolutionarily radical substitutions may be associated with functional disruption, the functional consequences of mutations are strongly dependent on their location within protein domains. Future work will extend this framework to incorporate location-specific mutational burden.

## Figures and Tables

**Table 1:** Combined summary statistics for global, PIK3CA, and TP53 missense mutation severity under codon-aware neutral models. ${\bar{B}}_{\mathrm{obs}}$ = observed mean BLOSUM; ${\bar{B}}_{\mathrm{neu}}$ = simulated neutral mean BLOSUM (mean ± SD); $f_{\mathrm{rad}}$ = radical-substitution fraction; $p_{\mathrm{rad}}$ = one-sided neutral-model Monte Carlo *p*-value for increased radical substitution.

Gene/Model	n	${\bar{B}}_{\mathrm{obs}}$	${\bar{B}}_{\mathrm{neu}}$	$f_{\mathrm{rad},\;\mathrm{obs}}$	$f_{\mathrm{rad},\;\mathrm{neu}}$	$p_{\mathrm{rad}}$
Global (Neutral Model)	204	–	−0.584±0.111	–	0.715±0.032	–
Global (Observed vs. Neutral)	204	−1.034	−1.398±0.100	0.848	0.896±0.021	0.989
PIK3CA	172	−0.034	−0.560±0.098	0.383	0.699±0.027	0.000
TP53	204	−1.034	−0.805±0.104	0.848	0.792±0.028	0.021

**Table 2:** Chi-square and likelihood-ratio G-tests evaluating whether observed mutation distributions across structural domains differ from codon-based neutral expectations. Both genes show extreme deviation from neutrality (*p*-values ≈0).

Gene	$\chi^{2}$ Statistic	$\chi^{2}$ *p*-Value	G Statistic	G *p*-Value
PIK3CA	212.0826	$7.37\times 10^{-44}$	246.7879	0.0
TP53	233.4057	$1.99\times 10^{-48}$	293.1543	0.0

**Table 3:** Domain-level PIK3CA missense mutation burden and BLOSUM severity compared to a codon-aware neutral model.

Domain	Neutral Frac	Observed Frac	Enrichment ($\frac{\mathrm{obs}}{\mathrm{neu}}$)	Neutral Mean	Observed Mean
ABD	0.0891	0.0170	0.19	−0.535	−0.167
C2	0.1509	0.1048	0.69	−0.726	−0.243
Helical	0.1666	0.3824	2.29	−0.492	0.778
Kinase	0.3402	0.4674	1.37	−0.350	−0.236
Other	0.1672	0.0283	0.17	−0.729	−0.600

**Table 4:** Domain-level TP53 missense mutation burden and BLOSUM severity compared to a codon-aware neutral model.

Domain	Neutral Frac	Observed Frac	Enrichment ($\frac{\mathrm{obs}}{\mathrm{neu}}$)	Neutral Mean	Observed Mean
CTD	0.0920	0.0000	0.00	−0.597	0.000
DBD	0.4487	0.9804	2.18	−0.772	−1.030
OD	0.1197	0.0147	0.12	−0.660	−2.000
Other	0.0702	0.0000	0.00	−0.845	0.000
PRD	0.1139	0.0000	0.00	−1.200	0.000

## Data Availability

The datasets and diagnostic slides are publicly available and anonymized in TCGA-BRCA at 10.7937/K9/TCIA.2016.AB2NAZRP, accessed on 28 December 2025. All code is available on GitHub (https://github.com/Alejandro21236/GenBlosum-WXSQCHEM), accessed on 28 December 2025. All datasets are public and anonymized.
